# Generation of the ICGi019-B-1 and ICGi019-B-2 lines via correction of the p.Met659Ile (c.1977G>A) variant in MYH7 of patient-specific induced pluripotent stem cells using CRISPR/Cas9

**DOI:** 10.18699/vjgb-25-38

**Published:** 2025-06

**Authors:** A.E. Shulgina, S.V. Pavlova, J.M. Minina, S.M. Zakian, E.V. Dementyeva

**Affiliations:** Institute of Cytology and Genetics of the Siberian Branch of the Russian Academy of Sciences, Novosibirsk, Russia; Institute of Cytology and Genetics of the Siberian Branch of the Russian Academy of Sciences, Novosibirsk, Russia; Institute of Cytology and Genetics of the Siberian Branch of the Russian Academy of Sciences, Novosibirsk, Russia; Institute of Cytology and Genetics of the Siberian Branch of the Russian Academy of Sciences, Novosibirsk, Russia; Institute of Cytology and Genetics of the Siberian Branch of the Russian Academy of Sciences, Novosibirsk, Russia

**Keywords:** hypertrophic cardiomyopathy, variants of unknown significance, induced pluripotent stem cells, CRISPR/Cas9, гипертрофическая кардиомиопатия, варианты с неясным клиническим значением, индуцированные плюрипотентные стволовые клетки, CRISPR/Cas9

## Abstract

The problem of interpretation of the genetic data from patients with inherited cardiovascular diseases still remains relevant. To date, the clinical significance of approximately 40 % of variants in genes associated with inherited cardiovascular diseases is uncertain, which requires new approaches to the assessment of their pathogenetic contribution. A combination of the induced pluripotent stem cell (iPSC) technology and editing the iPSC genome with CRISPR/Cas9 is thought to be the most promising tool for clarifying variant pathogenicity. A variant of unknown significance in MYH7, p.Met659Ile (c.1977G>A), was previously identified in several genetic screenings of hypertrophic cardiomyopathy patients. In this study, the single nucleotide substitution was corrected with CRISPR/Cas9 in iPSCs generated from a carrier of the variant. As a result, two iPSC lines (ICGi019-B-1 and ICGi019-B-2) were generated and characterized using a standard set of methods. The iPSC lines with the corrected p.Met659Ile (c.1977G>A) variant in MYH7 possessed a morphology characteristic of human pluripotent cells, expressed markers of the pluripotent state (the OCT4, SOX2, NANOG transcription factors and SSEA-4 surface antigen), were able to give rise to derivatives of three germ layers during spontaneous differentiation, and retained a normal karyotype (46,XY). No CRISPR/Cas9 off-target activity was found in the ICGi019-B-1 and ICGi019-B-2 iPSC lines. The maintenance of the pluripotent state and normal karyotype and the absence of CRISPR/Cas9 off-target activity in the iPSC lines with the corrected p.Met659Ile (c.1977G>A) variant in MYH7 allow using the iPSC lines as an isogenic control for further studies of the variant pathogenicity and its impact on the hypertrophic cardiomyopathy development.

## Abstract (ru)

Проблема интерпретации результатов генетического анализа пациентов, страдающих наследственными сердечно-сосудистыми заболеваниями, по-прежнему сохраняет свою актуальность. На сегодняшний день клиническое значение около 40 % вариантов в генах, ассоциированных с наследственными сердечно-сосудистыми заболеваниями, остается неясным, что приводит к необходимости использования новых подходов для оценки патогенетического вклада этих вариантов. Совместное применение технологии индуцированных плюрипотентных стволовых клеток и редактирования их генома с помощью системы CRISPR/ Cas9 считается наиболее перспективным способом выяснения патогенности генетических вариантов. Ранее в нескольких генетических скринингах пациентов с гипертрофической кардиомиопатией был выявлен вариант с неясным клиническим значением в гене MYH7, p.Met659Ile (c.1977G>A). В настоящем исследовании данная однонуклеотидная замена с помощью системы CRISPR/Cas9 была исправлена в индуцированных плюрипотентных стволовых клетках, полученных от носителя этого генетического варианта. В результате получены и охарактеризованы с использованием стандартного набора методов две линии индуцированных плюрипотентных стволовых клеток (ICGi019-B-1 и ICGi019-B-2). Линии индуцированных плюрипотентных стволовых клеток с исправленным вариантом p.Met659Ile (c.1977G>A) в гене MYH7 имели характерную для плюрипотентных клеток человека морфологию, экспрессировали маркеры плюрипотентного состояния (транскрипционные факторы OCT4, SOX2, NANOG и поверхностный антиген SSEA-4), были способны давать производные трех зародышевых листков при спонтанной дифференцировке и сохраняли нормальный кариотип (46,XY). В линиях индуцированных плюрипотентных стволовых клеток ICGi019-B-1 и ICGi019-B-2 не обнаружено нецелевой активности системы CRISPR/Cas9. Поддержание плюрипотентного состояния и нормального кариотипа, а также отсутствие нецелевой активности системы CRISPR/Cas9 в линиях индуцированных плюрипотентных стволовых клеток с исправленным вариантом p.Met659Ile (c.1977G>A) в гене MYH7 позволят использовать полученные линии в качестве изогенного контроля для дальнейших исследований патогенности данного генетического варианта и его влияния на развитие гипертрофической кардиомиопатии.

## Introduction

Generation of induced pluripotent stem cells (iPSCs) and their subsequent differentiation into cardiomyocytes is an important tool for modeling, studying, and developing therapy methods for inherited cardiovascular diseases (Parrotta et al., 2020; Funakoshi, Yoshida, 2021; Gähwiler et al., 2021). A combined use of the iPSC-based technology and genome editing methods, e. g. CRISPR/Cas9, also allows generating the so-called isogenic iPSCs by introducing a certain variant into iPSCs of a healthy donor or its correction in patient-specific iPSCs. Examination of cardiomyocytes derived from the isogenic iPSCs can overcome the challenge caused by numerous variants of unknown significance found in genetic screenings of patients with cardiovascular diseases (Guo H. et al., 2021).

Hypertrophic cardiomyopathy (HCM) is one of the most widespread inherited cardiovascular pathologies (overall prevalence is 0.2 %). The disease manifestations include an asymmetric thickness of the left ventricular walls and the interventricular septum, left ventricular outflow tract obstruction, progressive heart failure, and a high risk of atrial or ventricular arrhythmias and sudden cardiac death (Geske et al., 2018). HCM-causing variants can be found in genes encoding proteins involved in sarcomere functioning and regulation of calcium homeostasis. There are also HCM phenocopies that are due to variants in genes associated with metabolic disorders, neuromuscular diseases, and RASopathies. However, the majority of HCM-causing variants (about 80 %) have been found in MYH7 and MYBPC3, encoding sarcomere proteins: β-myosin heavy chain and myosin-binding protein C, respectively (Akhtar, Elliott, 2018; Pasipoularides, 2018).

A variant of unknown significance, p.Met659Ile (c.1977G>A, rs1241603111) in MYH7, was found in a number of genetic screenings of HCM patients (Richard et al., 2003; Bashyam et al., 2012; Dementyeva et al., 2020a). The single nucleotide substitution is a rare variant with no frequency in gnomAD v4.1.0 (https://gnomad.broadinstitute.org/) and is located in the actin-binding site of the myosin motor domain that is highly conservative in vertebrates (Hesaraki et al., 2022). The variant is predicted to be pathogenic by multiple in silico tools and AlphaMissense (Dementyeva et al., 2020a; Cheng et al., 2023; Pavlova et al., 2024). However, according to the ClinVar database (https://www.ncbi.nlm.nih.gov/clinvar), the available evidence is not sufficient to determine the role of the variant in HCM development. Therefore, functional studies are required to find out the pathogenicity of the variant.

In our previous study, CRISPR/Cas9 was used to introduce the variant into iPSCs of a healthy donor (Malakhova et al., 2020) and an iPSC line heterozygous at the single nucleotide substitution was generated. The cardiomyocytes derived from the iPSC line with the introduced p.Met659Ile (c.1977G>A) variant in MYH7 were characterized by an increased size, an elevated diastolic calcium level, changes in the expression of HCM-related genes, and a decrease in basal oxygen consumption rate compared to the isogenic control, which indicates the pathogenicity of the variant (Pavlova et al., 2024). However, it would be useful to verify the effects of the variant on the cardiomyocyte properties under another genetic background. This study was aimed at correction of the variant in an iPSC line derived earlier from an HCM patient carrying the p.Met659Ile (c.1977G>A) variant in MYH7 (Dementyeva et al., 2020b) and generation of the second panel of isogenic iPSC lines for studying the impact of the variant on HCM development.

## Materials and methods

iPSC lines used. ICGi019-B (https://hpscreg.eu/cell-line/ ICGi019-B), an iPSC line derived from an HCM patient who was a carrier of the p.Met659Ile (c.1977G>A) variant in MYH7 (Dementyeva et al., 2020b). ICGi022-A (https://hpscreg.eu/ cell-line/ICGi022-A), an iPSC line derived from a healthy donor (Malakhova et al., 2020).

iPSC cultivation. iPSC lines were cultured at 37 °C in 5 % CO2 on a layer of mitotically inactivated mouse embryonic fibroblasts (feeder) in KnockOut DMEM supplemented with 15 % KnockOut Serum Replacement, 0.1 mM MEM Non- Essential Amino Acids Solution, 1× penicillin-streptomycin, 1 mM GlutaMAX (all reagents – Thermo Fisher Scientific), 0.05 mM 2-mercaptoethanol (Amresco), and 10 ng/mL bFGF (SCI-store). The iPSC lines were passaged with TrypLE™ Express Enzyme (Thermo Fisher Scientific) at a ratio of 1:10 every 4–5 days.

Correction of the p.Met659Ile (c.1977G>A) variant in MYH7 of patient-specific iPSCs. 100 pmol of single-guide RNA (Synthego, Table 1) and 20 pmol of Cas9_NLS (NEB) were incubated for 20 min at room temperature. The ribonucleoprotein complexes, together with 300 ng of single-stranded donor oligonucleotide (Biolegio, Table 1), were electroporated into 1 × 105 iPSCs of the ICGi019-B line on a Neon Transfection System (Thermo Fisher Scientific), using the program: 1100 V, 30 ms, 1 time. The electroporated cells were transferred to a feeder layer in the iPSC medium without antibiotic and supplemented with 10 ng/ml Y-27632 (Sigma-Aldrich). 48 h later, the cells were subcloned into 96-well plates. iPSC clones were cultivated as described in the previous section.

**Table 1.**
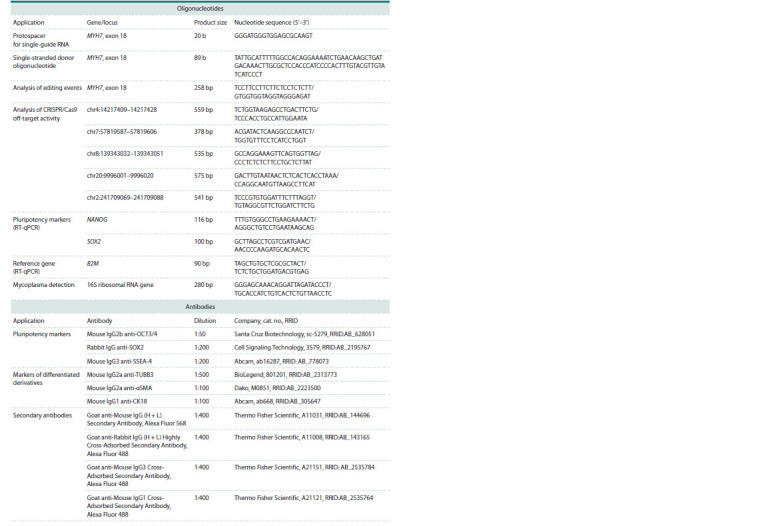
Oligonucleotides and antibodies used in this study

Analysis of editing events in MYH7 and CRISPR/Cas9 off-target activity. Genomic DNA was isolated from the iPSC clones using Wizard® Genomic DNA Purification Kit (Promega). Genomic DNA regions contained exon 18 of MYH7 or CRISPR/Cas9 off-target sites predicted using IDT (https:// www.idtdna.com/) were amplified by PCR with BioMaster HS-Taq PCR-Color (2×) (Biolabmix) on a T100 Thermal Cycler (Bio-Rad), using the program: 95 °С – 3 min; 35 cycles: 95 °С – 30 s, 62 °С – 30 s, 72 °С – 30–40 s; 72 °С – 5 min. The primers used are listed in Table 1. Sanger sequencing of the PCR products was performed using the Big Dye Terminator V. 3.1. Cycle Sequencing Kit (Applied Biosystems) and analysis was conducted at the SB RAS Genomics Core Facility (http://www.niboch.nsc.ru/doku.php/corefacility).

Karyotype analysis. iPSC lines were plated at a ratio of 1:4 on a 12-well plate 48 h before metaphase collection. Four different concentrations of Colcemide (from 25 to 50 ng/mL) were added 2.5 h before metaphase collection. Cells were disaggregated with TrypLE™ Express Enzyme (Thermo Fisher Scientific). Hypotonic treatment was conducted for 20 min at 37 °С in 0.28 % KCl. Cells were fixed in Carnoy’s solution (methanol–acetic acid 3:1) as described in (Sorogina et al., 2023). Karyotype of the iPSC lines was analyzed on Axioplan 2 (Zeiss) with the ISIS 5 program (MetaSystems). 50 metaphase plates were analyzed for the iPSC lines.

Spontaneous in vitro differentiation. iPSCs were treated for 40 min with 0.15 % Collagenase IV (Thermo Fisher Scientific). The resulting cell aggregates were transferred to Petri dishes coated with 1 % agarose and cultivated for 2 weeks in DMEM/F12 (1:1) medium supplemented with 15 % KnockOut Serum Replacement, 0.1 mM MEM Non-Essential Amino Acids Solution, 1× penicillin-streptomycin, 1 mM GlutaMAX (all reagents – Thermo Fisher Scientific). The embryoid bodies formed were plated on 8-well Chambered Coverglasses (Thermo Fisher Scientific) coated with Matrigel (Corning) and cultured for a week in the same medium. The medium was changed every 3 days. The differentiated derivatives were analyzed by immunofluorescence staining.

Immunofluorescence staining. iPSCs or their differentiated derivatives were fixed in 4 % paraformaldehyde (Sigma- Aldrich) for 10 min, permeabilized in 0.4 % Triton-Х100 (Sigma-Aldrich) for 10 min, incubated with 1 % bovine serum albumin (VWR) for 30 min (all the steps were conducted at room temperature). In case of SSEA-4, cell treatment was carried out without the permeabilization step. Cells were incubated overnight at 4 °С with primary antibodies and for 1 h at room temperature with secondary antibodies. After each incubation with antibodies, the cells were washed with PBS twice for 15 min. The antibodies used are provided in Table 1. Nuclei were counterstained with DAPI (Sigma-Aldrich). Immunofluorescence staining was analyzed on a Nikon Eclipse Ti-E microscope with NIS Elements Advanced Research software version 4.30 (Nikon).

RT-qPCR. RNA was isolated from iPSC lines with TRIzol Reagent and treated using the Invitrogen™ DNA-free™ DNA Removal Kit (all reagents – Thermo Fisher Scientific). Reverse transcription of 1 μg of RNA was conducted with M-MuLV reverse transcriptase (Biolabmix). RT-qPCR was performed with BioMaster HS-qPCR SYBR Blue 2× (Biolabmix) on a QuantStudio™ 5 Real-Time PCR System (Applied Biosystems), using the program: 95 °C – 5 min; 40 cycles: 95 °С – 10 s, 60 °С – 1 min. CT values were normalized by the ΔΔCT method (Livak, Schmittgen, 2001) using B2M as a reference gene. The primers used are listed in Table 1.

Mycoplasma detection. Mycoplasma contamination in iPSC lines was detected by PCR with BioMaster HS-Taq PCRColor (2×) (Biolabmix) on a T100 Thermal Cycler (Bio-Rad), using the program: 95 °C – 3 min; 35 cycles: 95 °C – 15 s, 67 °C – 15 s, 72 °C – 20 s; 72 °C – 5 min. The primers used are listed in Table 1.

## Results

**Fig. 1.**
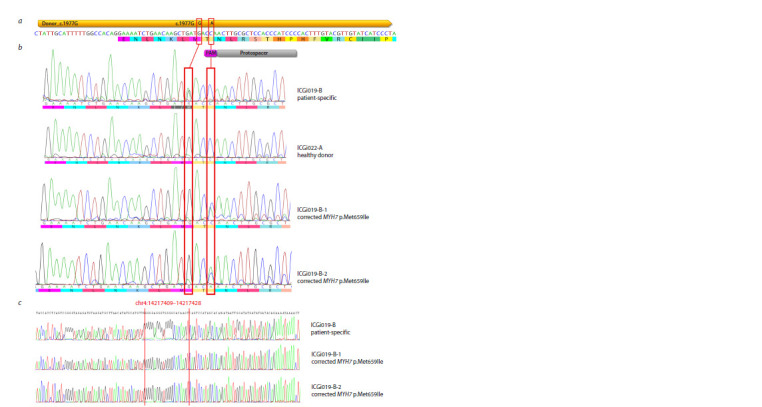
Correction of the p.Met659Ile (c.1977G>A) variant in MYH7 of the patient-specific iPSCs using CRISPR/Cas9. a – design of single-guide RNA and single-stranded donor oligonucleotide for MYH7 editing. The nucleotide sequence of a fragment of intron 17 and exon 18 is given. The positions of the protospacer for the single-guide RNA, PAM, and the single-stranded donor oligonucleotide are indicated in grey, magenta, and yellow, respectively. The target substitution and synonymous substitution in PAM are shown with red rectangles; b – an example of two iPSC clones with the corrected p.Met659Ile (c.1977G>A) variant in MYH7 (ICGi019-B-1 and ICGi019-B-2). Nucleotide sequences of the same region in the patient-specific iPSC line (ICGi019-B) and the iPSC line of the healthy donor (ICGi022-A) are provided for comparison. The target substitution and synonymous substitution in PAM are shown with red rectangles; c – absence of CRISPR/Cas9 off-target activity at one of the predicted CRISPR/Cas9 off-target sites in the iPSC lines with the corrected p.Met659Ile (c.1977G>A) variant in MYH7 (ICGi019-B-1 and ICGi019-B-2). The nucleotide sequence of the same region in the patient-specific iPSC line used for MYH7 editing (ICGi019-B) is given for comparison. The CRISPR/Cas9 off-target site and its positions in the human genome (hg38) are indicated in red.

The p.Met659Ile (c.1977G>A) variant in MYH7 was corrected in the patient-specific ICGi019-B iPSC line via introduction of a double-strand break with CRISPR/Cas9 and subsequent homology-directed repair with single-stranded donor oligonucleotide. The protospacer for the single-guide RNA and Protospacer Adjacent Motif (PAM) were designed to be located as close as possible to the target substitution and to introduce a synonymous substitution in PAM to protect MYH7 from repetitive editing (Fig. 1a). Low off-target activity of the selected single-guide RNA was confirmed using Benchling (https://www.benchling.com/) and IDT (https://www.idtdna. com/). The single-stranded donor oligonucleotide was chosen to correspond to the reference nucleotide sequence of a part of MYH7 intron 17 and exon 18 and contained the synonymous substitution (Fig. 1a).

CRISPR/Cas9 ribonucleoprotein complexes consisting of single-guide RNA and Cas9_NLS, together with the singlestranded donor oligonucleotide, were electroporated into cells of the ICGi019-B line. 84 iPSC clones were generated and analyzed using Sanger sequencing. In 71 (84.52 %) iPSC clones, editing events in MYH7 exon 18 were found. Nonhomologous end joining (indels) occurred in 54 (64.29 %) of the iPSC clones. Homology-directed repair with the singlestranded donor oligonucleotide accompanied by p.Met659Ile (c.1977G>A) variant correction was detected in 17 (20.23 %) iPSC clones. However, 9 iPSC clones with the homologydirected repair in the mutant allele also demonstrated off-target editing events (indels) in the second allele. Thus, 8 iPSC clones with the corrected p.Met659Ile (c.1977G>A) variant in MYH7 have been generated (Fig. 1b).

**Fig. 2.**
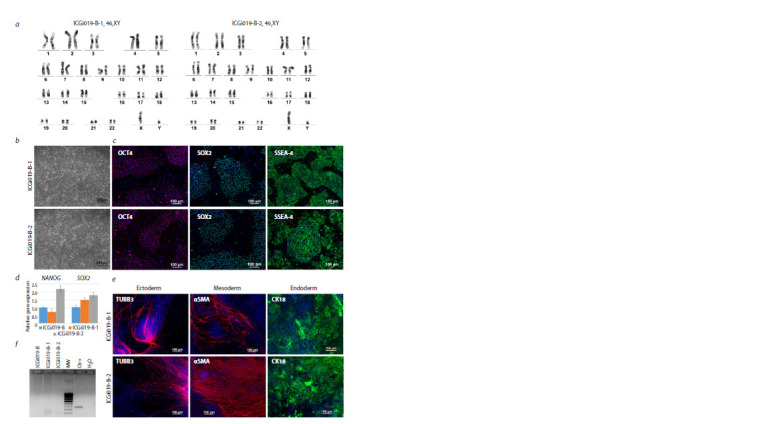
Characterization of the iPSC lines with the corrected p.Met659Ile (c.1977G>A) variant in MYH7. a – karyotype of the iPSC lines with the corrected p.Met659Ile (c.1977G>A) variant in MYH7 (ICGi019-B-1 and ICGi019-B-2); b – morphology of the ICGi019-B-1 and ICGi019-B-2 iPSC lines. Scale bar 250 μm; c – expression of the OCT4 and SOX2 transcription factors and SSEA-4 surface antigen in the ICGi019-B-1 and ICGi019-B-2 iPSC lines. Scale bar 100 μm; d – expression of pluripotency genes, NANOG and SOX2, in the ICGi019-B-1 and ICGi019-B-2 iPSC lines in comparison with the original patient-specific ICGi019-B line. Data are presented as mean ± SEM; e – capacity of the ICGi019-B-1 and ICGi019-B-2 iPSC lines to be differentiated into derivatives of three germ layers: ectoderm (TUBB3, β3- tubulin), mesoderm (αSMA, smooth muscle α-actin), and endoderm (CK18, cytokeratin 18). Scale bar 100 μm; f – absence of mycoplasma contamination in the ICGi019-B-1 and ICGi019-B-2 iPSC lines. Ctr+, positive control for mycoplasma contamination, H2O, negative control for mycoplasma contamination

For use in fundamental and applied studies, iPSC lines have to match a number of criteria, including the normal karyotype. Karyotype analysis of the iPSC lines with the corrected p.Met659Ile (c.1977G>A) variant in MYH7 showed that two iPSC lines, ICGi019-B-1 and ICGi019-B-2, retained the normal number and structure of chromosomes – 46,XY (Fig. 2a). The ICGi019-B-1 and ICGi019-B-2 iPSC lines were chosen for further characterization. No CRISPR/Cas9 off-target activity was found after comparison of the nucleotide sequences of the top-five CRISPR/Cas9 off-target sites and their surroundings in the iPSC lines and the patient-specific ICGi019-B line used for MYH7 editing (Fig. 1c). Despite MYH7 editing, the ICGi019-B-1 and ICGi019-B-2 iPSC lines retained their pluripotent properties. The iPSC lines possessed morphology similar to that of human pluripotent stem cells (Fig. 2b) and were characterized by expression of the pluripotent state markers such as the OCT4 and SOX2 transcription factors and SSEA-4 surface antigen (Fig. 2c). The expression level of pluripotency genes, NANOG and SOX2, in the ICGi019-B-1 and ICGi019-B-2 iPSC lines was demonstrated to be comparable to that in the original patient specific iPSC line (Fig. 2d). The iPSC lines with the corrected p.Met659Ile (c.1977G>A) variant in MYH7 were capable to be differentiated into derivatives of three germ layers as was shown by expression of markers of ectoderm, mesoderm, and endoderm in cells obtained under spontaneous differentiation of the iPSC lines in embryoid bodies (Fig. 2e). The iPSC lines were also free from mycoplasma contamination (Fig. 2f ). The ICGi019-B-1 and ICGi019-B-2 iPSC lines were registered in the Human Pluripotent Stem Cell Registry (hPSCreg, https:// hpscreg.eu/). Their passport is provided in Table 2.

**Table 2.**
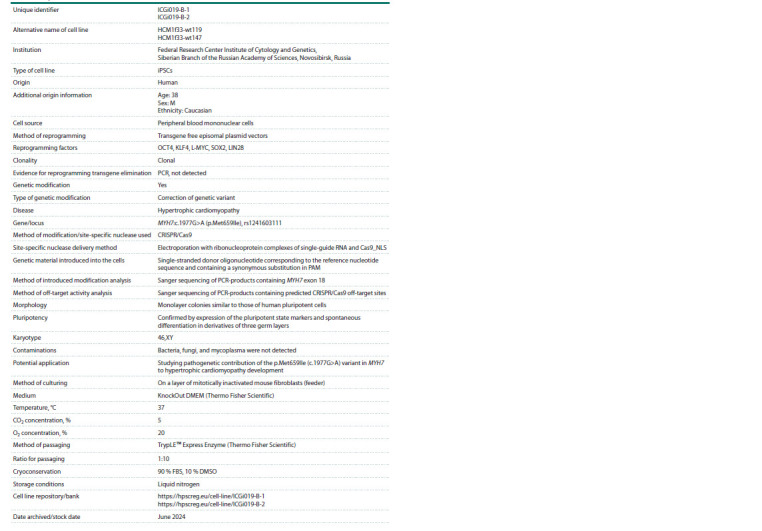
Passport of the ICGi019-B-1 and ICGi019-B-2 iPSC lines

## Discussion

iPSC editing with CRISPR/Cas9 has been successfully applied for studying HCM pathogenetic mechanisms caused by known pathogenic variants in sarcomere protein genes (Mosqueira et al., 2018; Smith et al., 2018; Wang et al., 2018; Cohn et al., 2019; Bhagwan et al., 2020; Shafaattalab et al., 2021; Chai et al., 2023; Escribá et al., 2023; Guo G. et al., 2024) and deciphering the pathogenicity of several variants of uncertain significance in HCM-associated genes (Ma et al., 2018; Pavlova et al., 2024).

This study was devoted to generating iPSC lines via correction of a variant of unknown significance, p.Met659Ile (c.1977G>A) in MYH7, in patient-specific iPSCs using CRISPR/Cas9. The variant is localized in the actin-binding site of the myosin motor domain where 37 variants have been described according to the ClinVar database. 12 variants are pathogenic and likely pathogenic whereas the remaining 25 (67.6 %) variants have uncertain significance or conflicting classifications of pathogenicity. This fact emphasizes the problem of interpretation of genetic data in clinical practice and makes examining the impact of variants of unknown significance in the functionally important actin-binding region much more relevant.

We previously introduced the p.Met659Ile (c.1977G>A) variant into MYH7 of the iPSCs from the healthy donor with CRISPR/Cas9 (Pavlova et al., 2024). Comparing the cardiomyocytes derived from the CRISPR/Cas9-edited iPSC line and its healthy isogenic control demonstrated that introduction of the variant resulted in appearance of HCM features such as an increased cardiomyocyte size, an elevated diastolic calcium level, a decreased basal oxygen consumption rate, and changes in expression pattern of HCM-related genes. These findings support the pathogenicity of the variant. However, validating the effects of the p.Met659Ile (c.1977G>A) variant in MYH7 under another genetic background could reinforce the conclusion on the variant clinical significance

The variant correction in the patient-specific iPSCs was performed using electroporation with CRISPR/Cas9 ribonucleoprotein complexes and single-stranded donor oligonucleotide. The method of CRISPR/Cas9 delivery was shown to cause a higher rate of editing events and reduced rate of CRISPR/ Cas9 off-target activity in comparison with CRISPR/ Cas9 delivery via plasmid transfection (Liang et al., 2015). To augment the efficiency of the editing process, we also used chemical modifications, 2′-O-methyl 3′ phosphorothioate and 3′ phosphorothioate bonds between the first three 5′ and 3′ terminal nucleotides, to protect the single-guide RNA and single-stranded donor oligonucleotide, respectively, from degradation and to stabilize the system (Hendel et al., 2015). As a result, a high percentage of the iPSC clones with editing events (84.52 %) and homology-directed repair (20.23 %) was observed after correction of the p.Met659Ile (c.1977G>A) variant in MYH7 of the patient-specific iPSCs. Moreover, no CRISPR/Cas9 off-target activity was revealed when analyzing the top-5 CRISPR/Cas9 off-target sites in the iPSC clones.

The iPSC lines with the corrected p.Met659Ile (c.1977G>A) variant in MYH7 (ICGi019-B-1 and ICGi019-B-2) matched all the criteria of human pluripotent stem cells. The iPSC lines had an appropriate morphology, expressed the main transcription factors and surface antigens characteristic of the pluripotent state, and gave rise to derivatives of three germ layers during spontaneous differentiation. This fact, together with the maintenance of the normal karyotype, makes the iPSC lines a good isogenic control for further verification of the variant pathogenicity and examination of HCM pathogenetic mechanisms triggered by the p.Met659Ile (c.1977G>A) variant in MYH7.

## Conclusion

Using CRISPR/Cas9, an HCM-associated variant of unknown significance, p.Met659Ile (c.1977G>A) in MYH7, was corrected in the patient-specific iPSCs. Eight iPSC lines with the corrected variant have been generated and two of the iPSC lines (ICGi019-B-1 and ICGi019-B-2) have been characterized in detail. The ICGi019-B-1 and ICGi019-B-2 iPSC lines retained the pluripotent status and normal karyotype and demonstrated no CRISPR/Cas9 off-target activity, which gives an opportunity to use the iPSC lines with the corrected p.Met659Ile (c.1977G>A) variant in MYH7 for studying the variant pathogenicity and role in HCM pathogenesis.

## Conflict of interest

The authors declare no conflict of interest.

## References

Akhtar M., Elliott P. The genetics of hypertrophic cardiomyopathy.
Glob Cardiol Sci Pract. 2018;2018(3):36. doi 10.21542/gcsp.
2018.36

Bashyam M.D., Purushotham G., Chaudhary A.K., Rao K.M., Acharya
V., Mohammad T.A., Nagarajaram H.A., Hariram V., Narasimhan
C. A low prevalence of MYH7/MYBPC3 mutations among
Familial Hypertrophic Cardiomyopathy patients in India. Mol Cell
Biochem. 2012;360(1-2):373-382. doi 10.1007/s11010-011-1077-x

Bhagwan J.R., Mosqueira D., Chairez-Cantu K., Mannhardt I., Bodbin
S.E., Bakar M., Smith J.G.W., Denning C. Isogenic models of
hypertrophic cardiomyopathy unveil differential phenotypes and
mechanism-driven therapeutics. J Mol Cell Cardiol. 2020;145:
43-53. doi 10.1016/j.yjmcc.2020.06.003

Chai A.C., Cui M., Chemello F., Li H., Chen K., Tan W., Atmanli A.,
McAnally J.R., Zhang Y., Xu L., Liu N., Bassel-Duby R., Olson E.N.
Base editing correction of hypertrophic cardiomyopathy in human
cardiomyocytes and humanized mice. Nat Med. 2023;29(2):401-
411. doi 10.1038/s41591-022-02176-5

Cheng J., Novati G., Pan J., Bycroft C., Žemgulyte A., Applebaum T.,
Pritzel A., … Senior A.W., Jumper J., Hassabis D., Kohli P., Avsec
Ž.
Accurate proteome-wide missense variant effect prediction with
AlphaMissense.
Science. 2023;381(6664):eadg7492. doi 10.1126/
science.adg7492

Cohn R., Thakar K., Lowe A., Ladha F.A., Pettinato A.M., Romano R.,
Meredith E., Chen Y.S., Atamanuk K., Huey B.D., Hinson J.T.
A contraction stress model of hypertrophic cardiomyopathy due to
sarcomere mutations. Stem Cell Rep. 2019;12(1):71-83. doi 10.1016/
j.stemcr.2018.11.015

Dementyeva E.V., Vyatkin Y.V., Kretov E.I., Elisaphenko E.A., Medvedev
S.P., Zakian S.M. Genetic analysis of patients with hypertrophic
cardiomyopathy. Genes Cells. 2020a;15(3):68-73. doi 10.23868/
202011011 (in Russian)

Dementyeva E.V., Kovalenko V.R., Zhiven M.K., Ustyantseva E.I.,
Kretov E.I., Vyatkin Y.V., Zakian S.M. Generation of two clonal
iPSC lines, ICGi019-A and ICGi019-B, by reprogramming peripheral
blood mononuclear cells of a patient suffering from hypertrophic
cardiomyopathy and carrying a heterozygous p.M659I
mutation in MYH7. Stem Cell Res. 2020b;46:101840. doi 10.1016/
j.scr.2020.101840

Escribá R., Larrañaga-Moreira J.M., Richaud-Patin Y., Pourchet L., Lazis
I., Jiménez-Delgado S., Morillas-García A., … de la Pompa J.L.,
Brugada R., Monserrat L., Barriales-Villa R., Raya A. iPSC-based
modeling of variable clinical presentation in hypertrophic cardiomyopathy. Circ Res. 2023;133(2):108-119. doi 10.1161/circresaha.
122.321951

Funakoshi S., Yoshida Y. Recent progress of iPSC technology in cardiac
diseases. Arch Toxicol. 2021;95(12):3633-3650. doi 10.1007/
s00204-021-03172-3

Gähwiler E.K.N., Motta S.E., Martin M., Nugraha B., Hoerstrup S.P.,
Emmert M.Y. Human iPSCs and genome editing technologies for
precision cardiovascular tissue engineering. Front Cell Dev Biol.
2021;9:639699. doi 10.3389/fcell.2021.639699

Geske J.B., Ommen S.R., Gersh B.J. Hypertrophic cardiomyopathy:
clinical update. JACC Heart Fail. 2018;6(5):364-375. doi 10.1016/
j.jchf.2018.02.010

Guo G., Wang L., Li X., Fu W., Cao J., Zhang J., Liu Y., … Liu G.,
Zhang Y., Dong J., Tao H., Zhao X. Enhanced myofilament calcium
sensitivity aggravates abnormal calcium handling and diastolic dysfunction
in patient-specific induced pluripotent stem cell-derived
cardiomyocytes with MYH7 mutation. Cell Calcium. 2024;117:
102822. doi 10.1016/j.ceca.2023.102822

Guo H., Liu L., Nishiga M., Cong L., Wu J.C. Deciphering pathogenicity
of variants of uncertain significance with CRISPR-edited
iPSCs.
Trends Genet. 2021;37(12):1109-1123. doi 10.1016/j.tig.
2021.08.009

Hendel A., Bak R.O., Clark J.T., Kennedy A.B., Ryan D.E., Roy S.,
Steinfeld I., … Bacchetta R., Tsalenko A., Dellinger D., Bruhn L.,
Porteus M.H. Chemically modified guide RNAs enhance CRISPRCas
genome editing in human primary cells. Nat Biotechnol. 2015;
33(9):985-989. doi 10.1038/nbt.3290

Hesaraki M., Bora U., Pahlavan S., Salehi N., Mousavi S.A.,
Barekat M., Rasouli S.J., Baharvand H., Ozhan G., Totonchi M.
A novel missense variant in actin binding domain of MYH7 is associated
with left ventricular noncompaction. Front Cardiovasc Med.
2022;9:839862. doi 10.3389/fcvm.2022.839862

Liang X., Potter J., Kumar S., Zou Y., Quintanilla R., Sridharan M.,
Carte J., Chen W., Roark N., Ranganathan S., Ravinder N., Chesnut
J.D. Rapid and highly efficient mammalian cell engineering
via Cas9 protein transfection. J Biotechnol. 2015;208:44-53. doi
10.1016/j.jbiotec.2015.04.024

Livak K.J., Schmittgen T.D. Analysis of relative gene expression data
using real-time quantitative PCR and the 2–ΔΔCT method. Methods.
2001;25(4):402-408. doi 10.1006/meth.2001.1262

Ma N., Zhang J.Z., Itzhaki I., Zhang S.L., Chen H., Haddad F., Kitani T.,
Wilson K.D., Tian L., Shrestha R., Wu H., Lam C.K., Sayed N.,
Wu J.C. Determining the pathogenicity of a genomic variant of uncertain
significance using CRISPR/Cas9 and human-induced pluripotent
stem cells. Circulation. 2018;138(23):2666-2681. doi 10.1161/
circulationaha.117.032273

Malakhova A.A., Grigor’eva E.V., Pavlova S.V., Malankhanova T.B.,
Valetdinova K.R., Vyatkin Y.V., Khabarova E.A., Rzaev J.A., Zakian
S.M., Medvedev S.P. Generation of induced pluripotent stem
cell lines ICGi021-A and ICGi022-A from peripheral blood mononuclear
cells of two healthy individuals from Siberian population.
Stem Cell Res. 2020;48:101952. doi 10.1016/j.scr.2020.101952

Mosqueira D., Mannhardt I., Bhagwan J.R., Lis-Slimak K., Katili P.,
Scott E., Hassan M., … Williams P.M., Gaffney D., Eschenhagen T.,
Hansen A., Denning C. CRISPR/Cas9 editing in human pluripotent
stem cell-cardiomyocytes highlights arrhythmias, hypocontractility,
and energy depletion as potential therapeutic targets for hypertrophic
cardiomyopathy. Eur Heart J. 2018;39(43):3879-3892. doi 10.1093/
eurheartj/ehy249

Parrotta E.I., Lucchino V., Scaramuzzino L., Scalise S., Cuda G. Modeling
cardiac disease mechanisms using induced pluripotent stem
cell-derived cardiomyocytes: progress, promises and challenges.
Int J Mol Sci. 2020;21(12):4354. doi 10.3390/ijms21124354

Pasipoularides A. Challenges and controversies in hypertrophic cardiomyopathy:
clinical, genomic and basic science perspectives.
Rev Esp Cardiol (Engl Ed). 2018;71(3):132-138. doi 10.1016/j.rec.
2017.07.003

Pavlova S.V., Shulgina A.E., Zakian S.M., Dementyeva E.V. Studying
pathogenetic contribution of a variant of unknown significance,
p.M659I (c.1977G>A) in MYH7, to the development of hypertrophic
cardiomyopathy using CRISPR/Cas9-engineered isogenic induced
pluripotent stem cells. Int J Mol Sci. 2024;25(16):8695. doi
10.3390/ijms25168695

Richard P., Charron P., Carrier L., Ledeuil C., Cheav T., Pichereau C.,
Benaiche A., … Desnos M., Schwartz K., Hainque B., Komajda M.,
EUROGENE Heart Failure Project. Hypertrophic cardiomyopathy:
distribution of disease genes, spectrum of mutations, and implications
for a molecular diagnosis strategy. Circulation. 2003;107(17):
2227-2232. doi 10.1161/01.CIR.0000066323.15244.54

Shafaattalab S., Li A.Y., Gunawan M.G., Kim B., Jayousi F., Maaref Y.,
Song Z., Weiss J.N., Solaro R.J., Qu Z., Tibbits G.F. Mechanisms
of arrhythmogenicity of hypertrophic cardiomyopathy-associated
troponin T (TNNT2) variant I79N. Front Cell Dev Biol. 2021;9:
787581. doi 10.3389/fcell.2021.787581

Smith J.G.W., Owen T., Bhagwan J.R., Mosqueira D., Scott E.,
Mannhardt I., Patel A., Barriales-Villa R., Monserrat L., Hansen A.,
Eschenhagen T., Harding S.E., Marston S., Denning C. Isogenic
pairs of hiPSC-CMs with hypertrophic cardiomyopathy/LVNC-associated
ACTC1 E99K mutation unveil differential functional deficits.
Stem Cell Rep. 2018;11(5):1226-1243. doi 10.1016/j.stemcr.
2018.10.006

Sorogina D.A., Grigor’eva E.V., Malakhova A.A., Pavlova S.V.,
Medvedev S.P., Vyatkin Y.V., Khabarova E.A., Rzaev J.A., Zakian
S.M. Creation of induced pluripotent stem cells ICGi044-B and
ICGi044- C using reprogramming of peripheral blood mononuclear
cells of a patient with Parkinson’s disease associated with с.1492T>G
mutation in the GLUD2 gene. Russ J Dev Biol. 2023;54(1):104-111.
doi 10.1134/S1062360423010125

Wang L., Kim K., Parikh S., Cadar A.G., Bersell K.R., He H., Pinto
J.R., Kryshtal D.O., Knollmann B.C. Hypertrophic cardiomyopathy-
linked mutation in troponin T causes myofibrillar disarray
and pro-arrhythmic action potential changes in human iPSC cardiomyocytes.
J Mol Cell Cardiol. 2018;114:320-327. doi 10.1016/
j.yjmcc.2017.12.002

